# Co-cultures with integrated in situ product removal for lactate-based propionic acid production

**DOI:** 10.1007/s00449-020-02300-0

**Published:** 2020-02-13

**Authors:** Ludwig Selder, Wael Sabra, Nikolai Jürgensen, Alagappan Lakshmanan, An-Ping Zeng

**Affiliations:** Institute of Bioprocess and Biosystems Engineering, Hamburg University of Technology, 21073 Hamburg, Germany

**Keywords:** In situ product removal, Co-culture, Propionic acid, Electrodialysis

## Abstract

Propionic acid (PA) is a valuable organic acid for the food and feed industry, but no bioproduction at industrial scale exists so far. As product inhibition is a major burden for bioprocesses producing organic acids, in situ product removal (ISPR) is desirable. Here, we demonstrate a new strategy to produce PA with a co-culture coupled with ISPR using electrodialysis. Specifically, Bacillus coagulans first produces lactic acid (LA) from sugar(s) and LA is converted to PA using Veillonella criceti. Applying ISPR to the mentioned co-culture, the specific PA yield was increased from 0.35 to 0.39 g g^−1^ compared to no ISPR usage. Furthermore, the productivity was increased from 0.63 to 0.7 g L^−1^ h^−1^ by applying ISPR. Additionally, it was shown that co-consumption of xylose and glucose led to a higher PA productivity of 0.73 g L^−1^ h^−1^, although PA yield was only increased slightly up to 0.36 g g^−1^.

## Introduction

Propionic acid (PA) is an important compound used in a variety of industries, e.g., for the production of food, polymers and pharmaceuticals [1, 38]. However, up to now, the vast majority of PA is produced via petrochemical processes, namely the Reppe and Larsson process [15]. To steer the chemical industry towards sustainable production routes, biotechnological processes have been studied to produce PA from bio-based resources [16; 35]. So far, the biotechnological route cannot compete economically with the petrochemical production process, due to limited yield, productivity, and high costs of product recovery when using bio-based substrates. Limitations during fermentation for bio-based PA occur mainly due to product inhibition and the generation of side products such as acetic acid (AA) and succinic acid (SA) [7, 15, 30]. The vast majority of studies concerning microbial PA production have been conducted with bacteria of the *Propionibacteria* species. Several strains such as *Propionibacterium acidipropionici*, *P. shermanii*,and *P. freudenreichii* have been investigated and bear the potential of reliable PA producers [12, 31–33]. However, product inhibition as well as side product formation are still the major challenges to overcome to reach a desired product titer of at least 100 g L^−1^ making a fermentative approach economic viable [15, 26]. Furthermore, these processes depend on glucose or glycerol as substrates and have a broad product spectrum, meaning that substrate is wasted for byproducts. A potential solution to this problem is the use of lactate as an intermediate substrate to produce PA and the application of ISPR to tackle the problem of product inhibition.

### Lactate as a platform substrate

Lactate is an important building block in the chemical industry with a well-established bio-based production process. Its production can be conducted relatively cheap and with economically viable product titers, productivities, and yields. A broad range of studies have been conducted with different substrates and operation modes with the highest product titer reported at 231 g L^−1^ by *R. oryzae* in fed-batch mode with glucose as substrate [34]. Various chemicals can be produced starting from bio-based lactate either via chemical catalysis [11] or directly by microorganisms which are able to utilize lactate as a substrate for growth such as *Veillonella* spp. [4, 5, 28].

*Bacillus coagulans* is an advantageous lactate producer as it possesses a broad pH spectrum ranging from 5.0 to 7.0 and a temperature tolerance up to 52 °C [37]. It is a Gram-positive and facultative anaerobe and able to grow in acidic environments generating high titers of l-lactate [14]. Of significant interest is the high selectivity which enables enantiomeric pure products. Zhang et al. [36] genetically modified a *B. coagulans* capable of producing d-lactate only, with an optical purity of 99.9%. Furthermore, the obtained yield of 0.98 g g^−1^, product titer of 145 g L^−1^, and possibility of unsterile fermentation prove the economic viability. Comparable results were achieved with a different *B. coagulans* strain selectively generating l-lactate with a final product titer of 182 g L^−1^ at a yield of 0.92 g g^−1^ [22]. Besides the high product titers and yields, *B. coagulans* tolerates a broad range of substrates such as corn steep powder, Jerusalem artichoke powder, wheat straw and lignocellulosic hydrolysates [2, 6, 30, 32]. These characteristics offer the chance to avoid using expensive substrates and instead using low-cost sustainable resources.

Co-cultures or cascade cultures can be used to exploit the availability of cheap substrates by converting them into lactate first which is then transformed into value-added chemicals. Sabra et al. [27] used a co-culture of *Veillonella criceti* and *Lactobacillus zeae* to produce PA from flour hydrolysate with a final product titer of 30 g L^−1^. The advantage of using *V. criceti* for the PA generation is the PA productivity of 0.39 g L^−1^ h^−1^. Productivities reached by *Propionibacterium* spp. are generally lower [27]. Apart from the lower productivity, the specific growth rate of *Propionibacterium acidipropionici* is ceasing with the generation of PA and reaching values close to 0 at concentrations above 10 g L^−1^ [20]. As *V. criceti* is not able to use glucose or xylose but lactate as an energy source, the carbon source has to be transformed to lactate first. However, during the fermentation for PA, the concentration of lactate has to be kept below 10 g L^−1^ to avoid substrate inhibition and PA has to be removed as product inhibition occurs [27].

### In situ product removal (ISPR) by electrodialysis

To overcome the aforementioned challenges, process intensification techniques can be applied, in this case in situ product removal (ISPR). There are several ISPR techniques suitable for the recovery of organic acids from fermentation, such as adsorption, extraction, nanofiltration/reverse osmosis, electrodialysis, and precipitation [21]. The problem arising when using filtration or adsorption methods is the fouling due to the cell attachment. In the case of precipitation, byproducts are generated in large quantities. Therefore, extraction and electrodialysis are the most common applications regarding ISPR. Extraction bears the obstacle of solvent toxicity as especially for the extraction of carboxylic acids, amine-based extractants diluted in long-chain hydrophobic organic compounds are the method of choice [17–19]. In this study, a modified electrodialysis, namely reversed electro-enhanced dialysis (REED), was used, selectively removing monovalent organic acids with an electric field as the driving force for ion migration. The REED technology has already been applied successfully as means for process intensification as it was shown that butyric acid production can be enhanced by applying REED [3]. The same setup was also studied to enhance the production of lactate [24]. In both cases, due to the REED usage and the internal membrane arrangement, operation malfunction because of fouling did not occur.

## Materials and methods

### Microorganisms and medium

*Bacillus coagulans* (DSMZ 2314) and *Veillonella criceti* (DSMZ 20734) were purchased from the German Collection of Microorganisms and Cell Cultures (DSMZ).

*Bacillus coagulans* was cultivated in a medium-containing: 13 g L^−1^ yeast extract, 10 g L^−1^ peptone, 5 g L^−1^ glucose, 1 g L^−1^ soluble starch, 5 g L^−1^ sodium chloride, and 3 g L^−1^ sodium acetate. Anaerobic conditions were ensured by flushing with pure nitrogen for 20 min at 80 °C. After cooling, 0.5 g L^−1^ cysteine-HCl was added.

*Veillonella criceti* was cultivated in a medium-containing: 2 g L^−1^ yeast extract, 2 g L^−1^ peptone, 15 g L^−1^ KH_2_PO_4_, 5 g L^−1^ K_2_HPO_4_, and 10 g L^−1^ potassium lactate. Anaerobic conditions were ensured by flushing with pure nitrogen for 20 min at 80 °C. After cooling, 0.5 g L^−1^ cysteine-HCl and a vitamin solution were added: 0.25 mg L^−1^ biotin, 0.01 mg L^−1^ folic acid, 2.5 mg L^−1^ Pyridoxin HCl, 50 mg L^−1^ Thiamine HCl, 50 mg L^−1^ Riboflavin, 2.5 mg L^−1^ Nicotinic acid, 2.5 mg L^−1^ Calcium Pantothenate, and 0.05 mg L^−1^ Vitamin B12. The vitamin solution was passed through a sterile filter (0.22 µm) before addition.

Co-cultures were performed with a media consisting of the following ingredients: 10 g L^−1^ yeast extract, 10 g L^−1^ peptone, 1.7 g L^−1^ (NH_4_)_2_SO_4_, 5 g L^−1^ (NH_4_)_2_PO_4_, 0.4 g L^−1^ MgSO_4_·7H_2_O, 15 g L^−1^ KH_2_PO_4_, 5 g L^−1^ K_2_HPO_4_, and 1.2 mL L^−1^ of the vitamin solution.

The glucose and xylose used in this study was generated from wheat straw and kindly provided by Prof. Irina Smirnova, Institute of Thermal Separation Processes, Hamburg University of Technology. Xylose solution was treated with activated carbon to remove acetate and analyzed using HPLC. No acetate was found in the xylose solution after this treatment. For glucose production, wheat straw was treated via the liquid hot-water process and supplied as a liquid biomass hydrolysate [25].

### Fermentation process

Cultivations were either performed in a stirred 2.4 L glass bioreactor (Bioengineering AG, Switzerland) or in a stirred 1.8 L glass bioreactor (DASGIP AG, Germany). The pH was controlled and corrected with 6 M KOH. The fermentation was initiated after the addition of 10% (v/v) inoculum from preculture. Samples were withdrawn sterile at regular intervals and the sugar concentration as well as the organic acid concentration were determined. Pre-cultures were grown in the respective medium under anaerobic conditions in glass vials with butyl rubber stoppers and aluminum clamps. Fermentations were performed at 37 °C and a controlled pH of 6.2.

Cascade cultures were performed using the fermentation broth of the *B. coagulans* fermentation as substrate. Prior to usage, cells were removed by centrifugation (Heraeus Megafuge) at 8500 rpm for 15 min.

Experiments to determine the inhibition by lactate were performed in anaerobic flasks (50 mL) with butyl rubber stops. Potassium lactate was added externally before inoculation.

### In situ product removal by electrodialysis

**Fig. 1:**
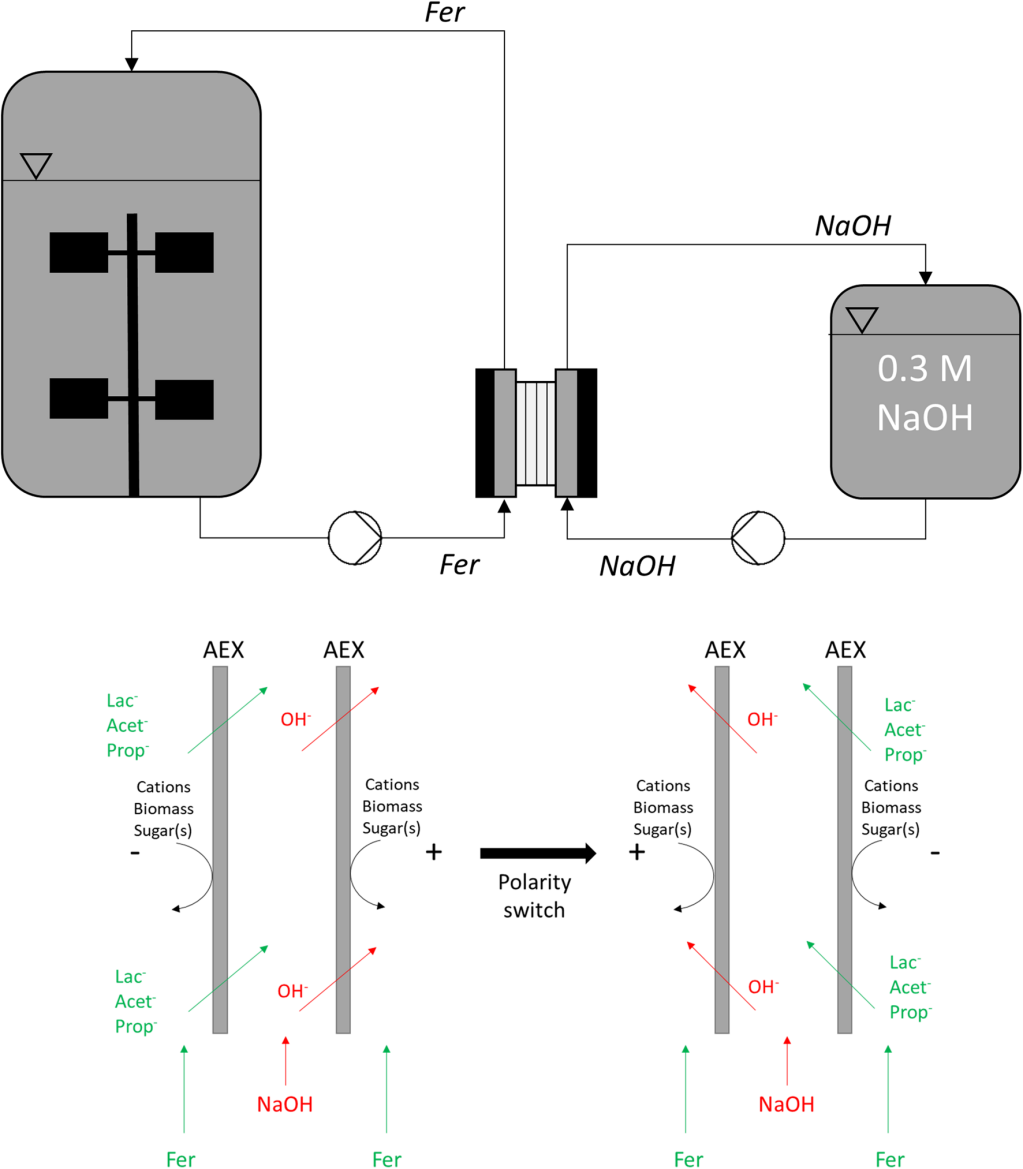
REED setup with in situ connection to bioreactor; Fer: stream of fermentation broth; NaOH: stream of NaOH; electrodes are flushed with electrolyte solution (not shown). Lower picture depicts the functionality of the polarity reversal which leads to a change in ion flux direction (*Lac* Lactate, *Prop* PA, *Acet* AA, *AEX* anion-exchange membrane)

The in situ product removal (ISPR) is accomplished via a modified electrodialysis unit. In this study, the REED unit is connected through a loop system with the bioreactor. In Fig. 1, it can be seen that the fermentation broth is cycled from the fermenter bottom subsequently through the electrodialysis unit and back into the fermenter at the top. In parallel, 0.3 M NaOH is pumped in the alternate flow channels of the REED stack from a reservoir in a circular fashion. The fermentation broth gets depleted of monovalent organic ions and the NaOH stream enriched in organic acids. The stack (PCCell, Germany) consists of ten anion-exchange (PC 100 D, PCCell) membranes, each with an effective membrane area of 0.0064 m^2^. These membranes can only be penetrated by monovalent negatively charged ions. The electrodes are manufactured of Pt/Ir enabling polarity switching. The polarity switch is enabled with a self-built device enabling switching at any time interval higher than 10 s. A maximum of 36 *V* and 4.5 *A* can be applied. The setup was operated in constant current mode. If not specifically mentioned, the current density was set at 400 A m^−2^ with a reversal of the polarity each 60 s. Electrode chamber flushing was performed with 0.25 M Na_2_SO_4_. Acids separated by the ISPR setup are gained as salts, namely Na^+^ as the counter stream of the fermentation broth is a 0.3 M NaOH solution. Detailed description of the setup as well as the anti-fouling mechanisms can be found in Prado-Rubio et al. [24]. Sterility was ensured by cycling 70% (v/v) ethanol for 30 min. Disinfection was performed by circling 70% (v/v) ethanol for 30 min, deionized water, and 5% (v/v) nitric acid for 30 min.

### Analytical methods

PA, AA, LA, xylose, and glucose were analyzed by HPLC (Kontron, Germany) with an Aminex HPX-87H column (300 × 7.8 mm) at 60 °C and UV (210 nm) and RI detectors. As mobile phase, 5 mM H_2_SO_4_ was used. All samples were filtered (0.22 µm) before HPLC analysis. The OD of the samples was measured using a UV/VIS V 1200 Spectrometer (VWR) at 600 nm.

## Results and discussion

### Monoculture of *B. coagulans*

**Fig. 2:**
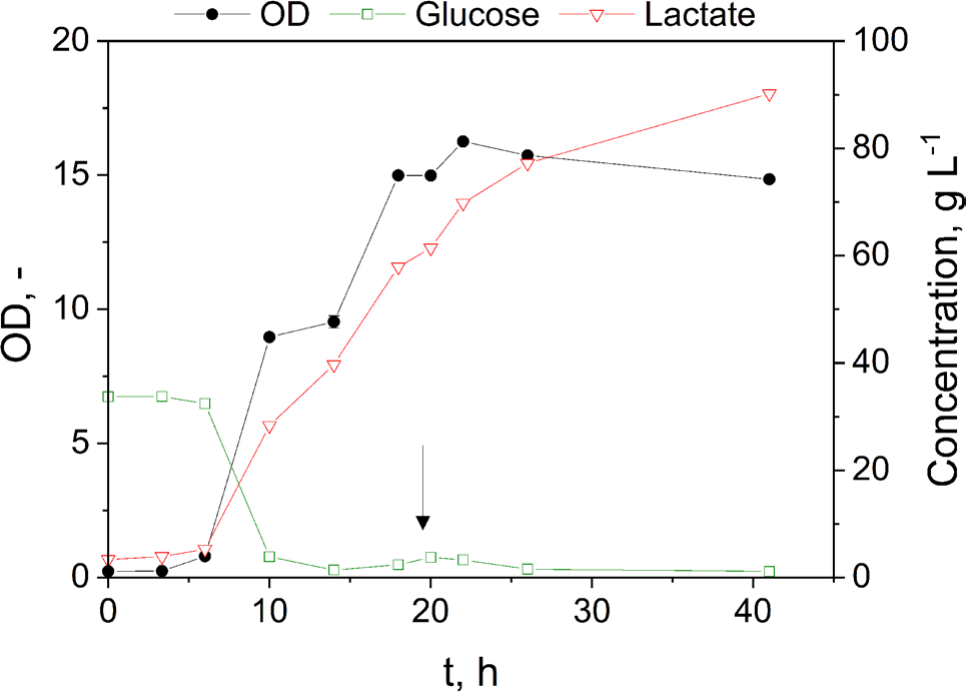
Glucose fermentation using *B. coagulans* as monoculture. The arrow indicates the addition of glucose

Figure 2 depicts the results of glucose fermentation using *B. coagulans* with the addition of glucose at a fermentation time of 20 h. Production of lactate exceeding the glucose consumption between 10 and 20 h of fermentation time is obviously due to use of complex medium components. The latter is necessary for cell growth, especially to provide the crucial nitrogen source [23]. The average lactate productivity peaked at 3.6 g L^−1^ h^−1^ during the exponential growth with a specific yield of 0.99 g lactate per g glucose with a final titer of 90.2 g L^−1^. As it can be determined from the specific yield, almost all of the substrate is converted into lactate, and only a small fraction is used for energy and biomass generation. For lactate generation, *B. coagulans* makes use of the pentose-phosphate pathway efficiently producing lactate without any byproducts [22]. Therefore, *B. coagulans* can serve as an optimal partner for lactate formation in co-cultures due to its efficient transformation of the substrate.

### Monoculture of *V. criceti*

**Fig. 3:**
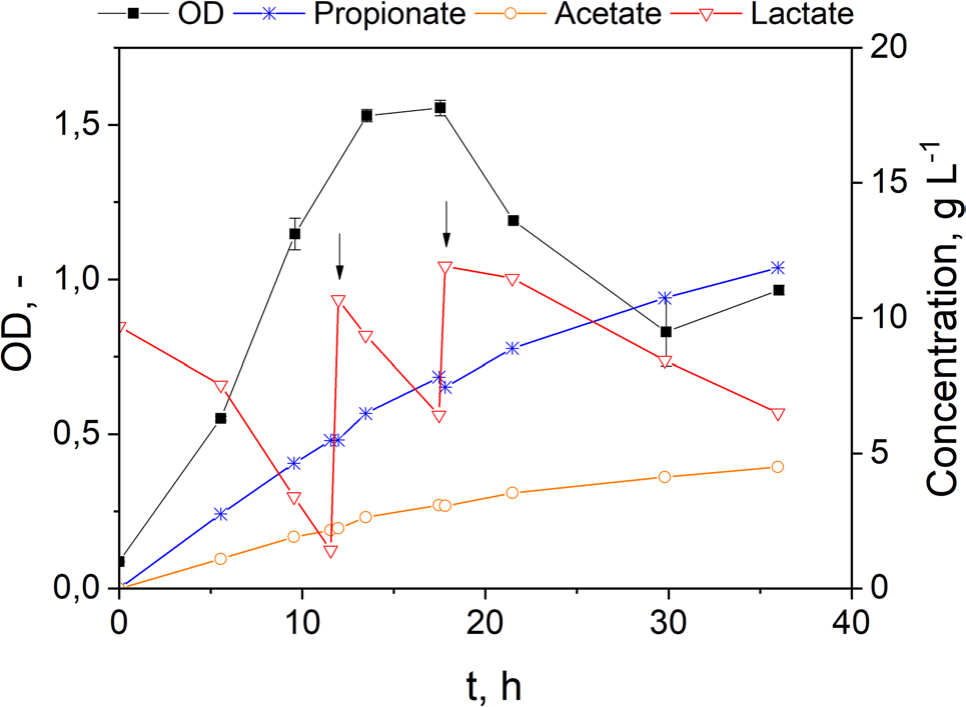
Repeated batch fermentation of lactate using *V. criceti*. Pulses of lactate from cultivation of *B. coagulans* were added at the indicated time points (black arrow)

Fermentation of lactate using *V. criceti* reached a maximum PA titer of 11.9 g L^−1^ with 4.5 g L^−1^ AA as a coproduct. The PA productivity reached 0.47 g L^−1^ h^−1^ with a yield of 0.49 g PA per g LA. However, it can be seen from the curve of OD development that above a PA concentration of 6.5 g L^−1^ growth ceases, and the OD drops, indicating lysis of cells. Several inhibition mechanisms have been proposed, though the exact mechanism of inhibition by organic acids towards microbial species remains unclear. Visible from Fig. 3 is the accompanied production of AA besides PA from lactate. According to Fuchs et al. [13], *Veillonella* spp. makes use of the available substrate either through the methyl-malonyl pathway generating PA via oxaloacetate or through the transformation of pyruvate to AA. Notable is the fact that *Veillonella* does not possess a transcarboxylase able to convert pyruvate to oxaloacetate directly, but a bypass route using a pyruvate carboxylase and a methyl-malonyl-CoA decarboxylase. The stoichiometry of the proposed pathway demands the generation of 2 mol PA and 1 mol of AA from 3 mol of lactate. The transformation of lactate to AA is used to generate ATP and NADH, while the PA route makes use of the generated NADH.

### Co-culture of *B. coagulans* and *V. criceti*

Co-cultures of *B. coagulans* and *V. criceti* were conducted in such a way that glucose was added in pulses to avoid overproduction of lactate diminishing the growth of *V. criceti*. Therefore, only that much glucose was added that the maximum lactate concentration reachable was always below 10 g L^−1^. Sabra et al. were able to show that strong inhibition occurs of *V. criceti* because of PA and LA. Above a PA or LA concentration above 10 g L^−1^, respectively, growth ceased rapidly.

*Veillonella criceti* was added 13.2 h after the inoculation of the bioreactor with *B. coagulans* to ensure the availability of lactate as a substrate generated from *B. coagulans* after its initial lag phase.

**Fig. 4:**
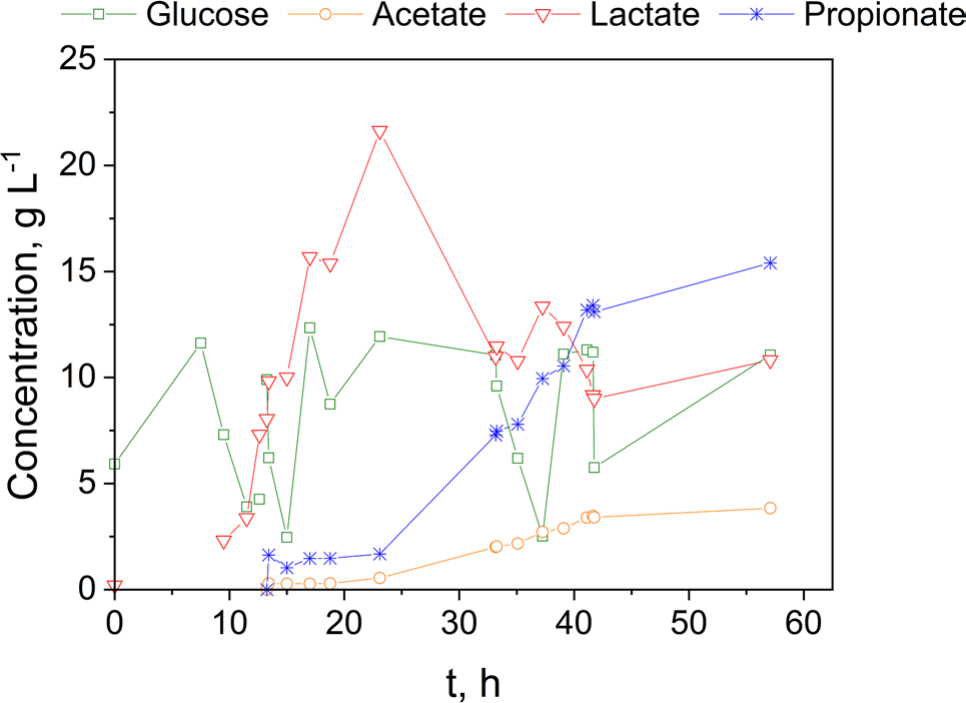
Co-culture of *B. coagulans* and *V. criceti* using glucose as substrate

As it can be seen from Fig. 4, the maximum product titer achieved by a co-culture was 15.4 g L^−1^ and 3.8 g L^−1^ for PA and AA, respectively. With the addition of the bacteria, glucose was added as well and almost immediately converted to lactate reaching a maximum value of 21.6 g L^−1^. The yield for PA from glucose is 0.35 g g^−1^ with a productivity of 0.63 g L^−1^ h^−1^. In this case, an inhibition of *V. criceti* does not seem to occur, as PA and AA are generated, although lactates exceeds the critical inhibition concentration and PA production is still detectable after the spike of LA at 22 h. This leads to the conclusion that the presence of *B.* *coagulans* might enable a higher tolerance of *V. criceti* towards PA and can be characterized as a mutualism culture [8].

### Co-culture of *B. coagulans* and *V. criceti* with in situ electrodialysis

**Fig. 5:**
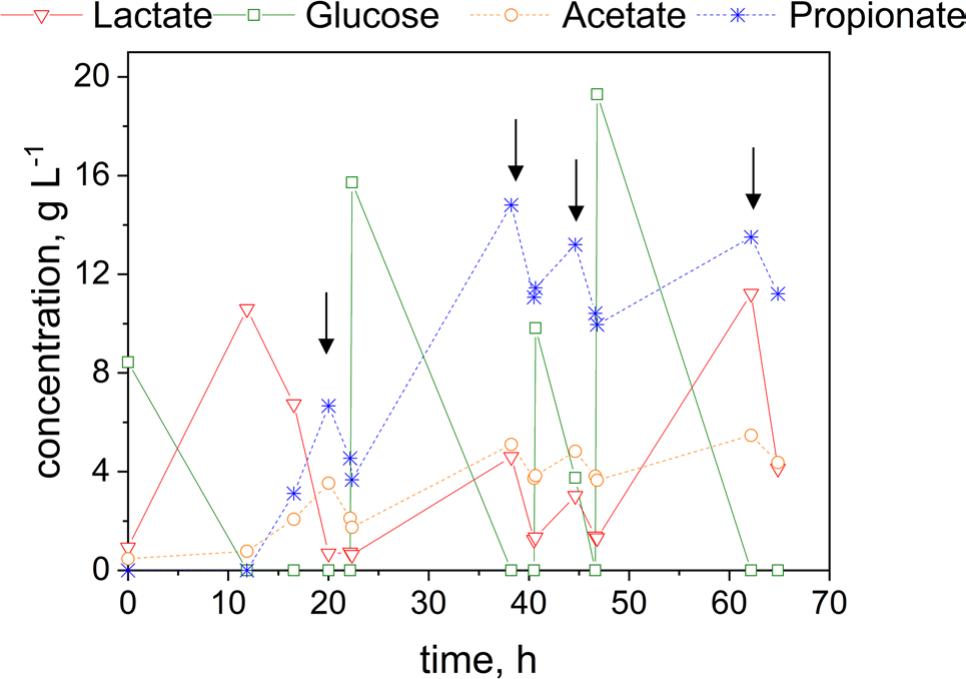
Fermentation of glucose using *B. coagulans* and *V. criceti* in co-culture. Black arrows indicate the start of an ISPR cycle (run time 2 h)

**Fig. 6:**
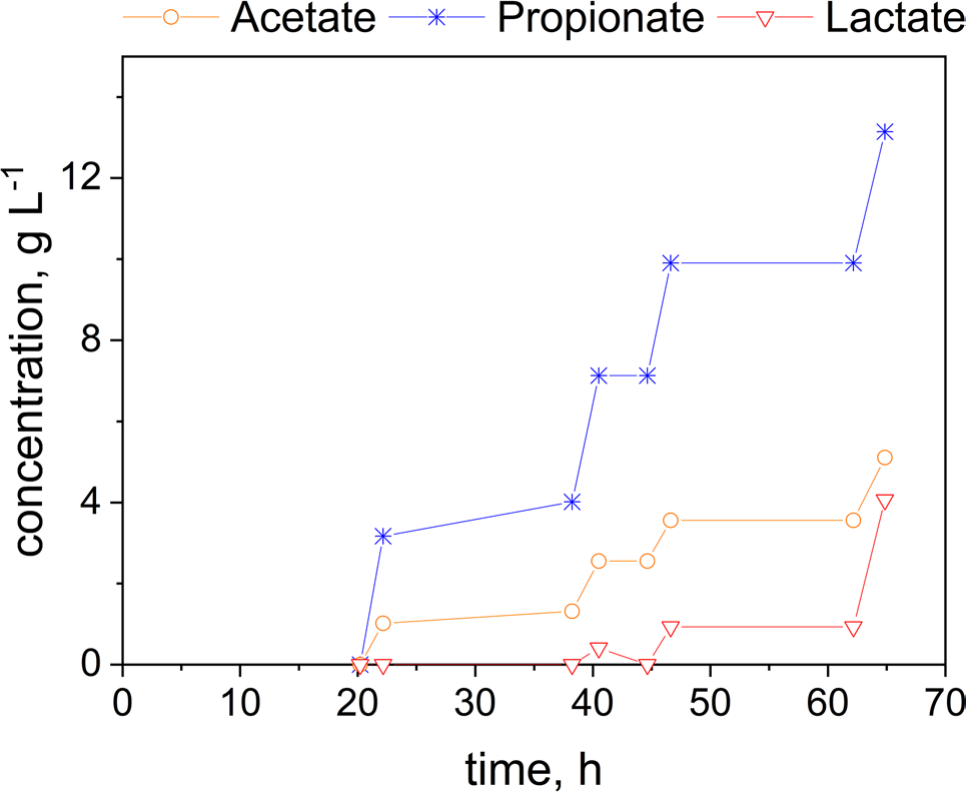
In situ product removal of PA, LA, and AA from fermentation (NaOH compartment)

To intensify the production process, the fermentation was coupled with an in situ REED setup as described earlier. This system enables the selective removal of monovalent ions and can theoretically allow infinite fermentation duration by continuously removing inhibiting substances accumulated inside the bioreactor. For the duration of the fermentation, glucose was added in pulses to avoid concentrations of lactate exceeding inhibitory levels. The REED setup was switched on when LA was consumed and PA and AA were generated. After the completion of an REED run, glucose was added again. Figure 5 shows the final product titer in the fermentation broth and Fig. 6 shows the final titer in the NaOH stream.

These results show the beneficial impact of the in situ product removal on the fermentation process. Besides an improved yield of 0.39 g g^−1^ PA, the productivity was enhanced, as well. Combining the concentrations reached in the electrodialysis, NaOH compartment and the fermentation 24.3 g L^−1^ of PA have been produced with a productivity of 0.70 g L^−1^ h^−1^. As it can be seen from Fig. 5, although inhibiting concentrations of PA are reached throughout the fermentation, PA is produced again after the removal of PA and AA. Figure 6 shows that the electrodialysis was applied four times totally, indicated by the increase in PA and AA in the dilute stream and the resulting depletion of these organic acids in the fermentation media. On average, the removal rate is 1.3 g L^−1^ h^−1^ for PA. As *V. criceti* is the bottleneck, due to its various inhibitions, in this bioprocess, close attention has to be paid to these bacteria. Therefore, the electrodialysis was only run for 2 h. By running the electrodialysis for a longer time span, more PA and AA would be removed. However, during the application of the REED, *V. criceti* is in a substrate-deficient state, as any lactate present in the fermentation media would also be removed with the REED setup. The curve of the LA concentration in Fig. 6 shows that almost no lactate is transported into the NaOH stream during the fermentation time between 20 and 60 h. Only at the end of the fermentation at 60 h when lactate accumulated in the bioreactor, LA was removed. After the complete consumption of LA by *V. criceti* at 20 h and the subsequent LA generation by the added glucose, no LA is removed, as all the LA is converted into PA and AA.

Comparing the results for the fermentation with and without ISPR it can be seen that the application of the REED system led to an increase in specific yield and productivity. The fermentation of glucose without ISPR reached a yield of 0.35 g g^−1^ with a productivity of 0.63 g L^−1^ h^−1^, while the REED integration resulted in a yield of 0.39 g g^−1^ with a productivity of 0.70 g L^−1^ h^−1^.

**Fig. 7:**
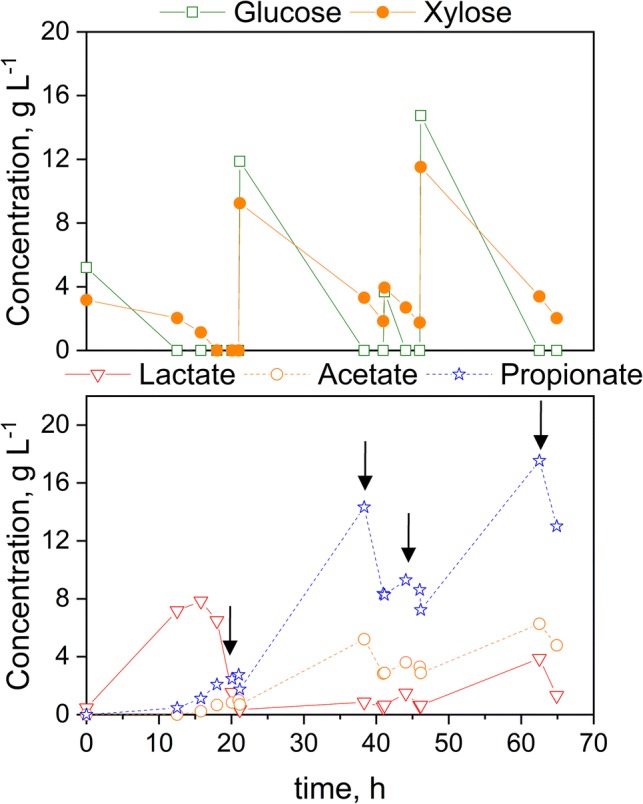
Fermentation of glucose and xylose using *B. coagulans* and *V. criceti*. Black arrows indicate the start of an ISPR cycle (run time 2 h)

**Fig. 8:**
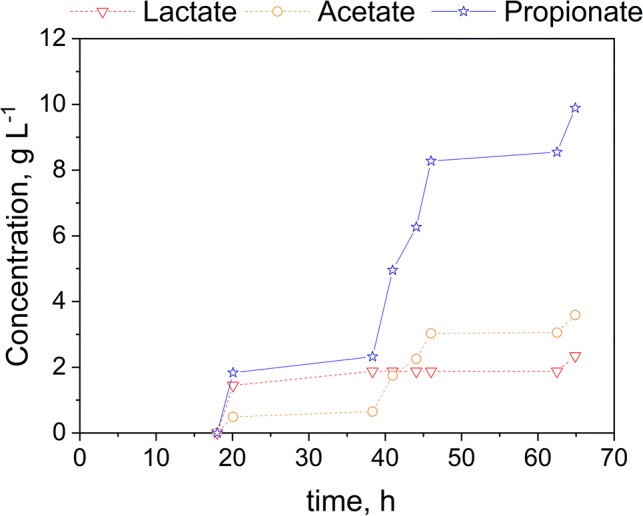
Concentration of organic acids in NaOH compartment of REED system

Additionally, fermentation of glucose and xylose as substrate was investigated. As it can be seen from Fig. 7, the substrate was fed in pulses. Glucose and xylose are consumed simultaneously and instantly with no initial lag phase. Furthermore, lactate is produced in parallel to glucose and xylose consumption without any delay. After each addition of the substrate mixture, glucose is consumed entirely each time, while xylose is only consumed entirely the first time and residual concentrations of xylose remain unused afterwards. Moreover, after the addition of *V. criceti* to the bioreactor at 11 h, the LA concentration drops, and subsequently, PA and AA are generated. It can be seen from the LA concentration course that the feeding combined with the REED works in a way that after 20 h no LA is removed from the bioreactor and the LA concentration can be maintained close to 0, while the PA and AA production is still ongoing. At 38 h and 62.5 h, the PA concentration is reaching inhibitory levels of 14.3 g L^−1^ and 17.5 g L^−1^, respectively. As observed above, growth and PA production is still ongoing, although inhibitory levels of PA were reached. From the lower diagram of Fig. 7, it can be concluded that the removal of PA and AA was successfully performed, as both concentrations are dropping throughout the application of the REED system and the concentrations of the respective organic acids are increasing in the NaOH compartment, as shown in Fig. 8. In total, it was possible to remove 9.9 g L^−1^ of PA and 3.6 g L^−1^ of AA. The increase in PA in the NaOH stream, between 44 and 46 h, is stemming from diffusion of these ions through the membranes due to the apparent concentration gradient, as no electrical field is applied. Combining REED and fermentation, a total amount of 23 g L^−1^ of PA were generated with a yield of 0.36 g PA per g substrate. Additionally, in this experiment, the highest PA production rate was reached at 0.73 g L^−1^ h^−1^. The residual concentrations of xylose may arise due to the fact that *B. coagulans* makes use of its preferred substrate, namely glucose and even repressing the utilization of xylose [29]. Therefore, it is possible to use a combination of sugars, such as lignocellulosic biomass for production of organic acids without the need of separation of different sugars before usage.

Relating the results of the fermentation with glucose and glucose and xylose as substrate, one can observe that the molar ratio of PA is enhanced when using mixed substrate at 2.21 mol PA per mol AA, while utilizing glucose at a ratio of 2.08 mol per mol only.

The increase in energy demand of the REED per g of PA, stated in Table 1, can be explained with the improved biomass generation when using a mixed substrate in comparison to glucose as the only substrate (results not shown). As more biomass is generated, the fouling on the membrane increases, and therefore, the resistance of the membrane stack increases, leading to higher voltages to uphold the current density. In comparison with previous studies, co-culture coupled with electrodialysis has proven to enhance PA productivity, as maximum productivities reached were 0.33 g L^−1^ h^−1^ when using flour hydrolysate as substrate and 0.61 g L^−1^ h^−1^ while using glucose as a substrate in a dialysis reactor in continuous mode [10, 27]. An overview of the generated experimental results can be found in Table 1.

**Table 1:** Overview of fermentation with different substrates and ISPR application

	Substrate	*Y*_P/S_, g g^−1^	*Q*_P,max_, g L^−1^ h^−1^	kWh g^−1^ PA
With ISPR	Glucose	0.39	0.70	0.03 ± 0.002
With ISPR	Glucose, xylose	0.36	0.73	0.043 ± 0.008
Without ISPR	Glucose	0.35	0.63	–

## Conclusion

In this study, it was successfully demonstrated that a co-culture of *B. coagulans* and *V. criceti* is able to produce PA from glucose and a combination of glucose and xylose via lactate. However, the product titer of propionate reached with this setup makes an industrial production not economically viable right now. An approach towards improving the product titer would be the engineering of the *Veillonella* strain to avoid the substrate and product inhibition. Further optimization has to be conducted regarding the feeding strategy. As the electrodialysis is also able to remove lactate, *Veillonella* is quickly in a substrate-deficient state during the operation of the REED. Therefore, it would be preferable to model and, in consequence, determine the optimal time point for the REED operation.

Starting from lactate as a platform intermediate gives the opportunity to establish novel processes based on lactate as the intermediate substrate when combining *B. coagulans* with a suitable partner. As *B. coagulans* transforms a broad variety of substrates to lactate only, it is possible to work with low-grade substrates and convert them to value-added products via a second bacterial species. Furthermore, by applying both measures, namely ISPR and co-cultures, a novel methodology towards bioprocess development can be taken. By approaching the process development according to the desired final product composition and concentration, a “process development by product design” concept is pursued. For example, for weed killing agents, a defined mixture of organic acids, such as acetic, lactic and propionic acid, is needed to ensure successful herbicidal properties [9]. Therefore, the bioprocess development is designed tailor-made according to the desired final organic acid concentration and composition, by choosing appropriate bacterial communities, the most suitable ISPR technique, and the economically most reasonable substrate. This bottom-up strategy is especially important in the transformation of the chemical industry from a petrochemical towards a bio-based one. As up-to-date biotechnological production routes often lack the economic profitability, the same product definitions have to be reached to compete with petrochemical routes.
